# Sensory receptor expansion and neural accommodation in butterfly color vision

**DOI:** 10.1101/2025.10.30.685642

**Published:** 2025-10-31

**Authors:** Ke Gao, Julia Ainsworth, Antoine Donati, Yunchong Zhao, Michelle Franc Ragsac, Cara Genduso, Zoie Andre, Andrew Tomlinson, Michael W. Perry

**Affiliations:** 1 Department of Cell & Developmental Biology, School of Biological Sciences, University of California San Diego, La Jolla, CA, 92093, USA; 2 Halicioğlu Data Science Institute, University of California San Diego, La Jolla, CA, 92093, USA; 3 Department of Biology, New York University, New York, NY, 10003, USA; 4 Department of Genetics and Development, College of Physicians and Surgeons, Columbia University, New York, NY, 10032, USA; 5 Zuckerman Mind Brain Behavior Institute, Columbia University, New York, NY, 10027, USA

## Abstract

The evolution of more complex brains required existing neurons and neural circuits to accommodate new inputs. The genetic and developmental mechanisms that enable such integration are largely unknown. Butterflies evolved more complex retinal mosaics through the addition of a second R7 color photoreceptor per ommatidium (unit eye). In *Drosophila*, the unique R7 makes a stochastic choice to express one of two opsin genes. In butterflies, the two R7s make two independent stochastic cell fate choices in each ommatidium, producing three ommatidial types instead of the two in flies. Here, we investigate the developmental basis of this change and how the butterfly brain accommodates an expansion in sensory receptor input. We first identified the developmental mechanisms that specify the second R7 cell. We then modified *Drosophila* retinas to have butterfly-like transcription factor expression, causing recruitment of an additional R7. The two R7s make independent stochastic choices, like butterflies, leading to three stochastically distributed ommatidial types instead of two. In *Drosophila*, each of the two subtypes of R7s connects to one of two types of Dm8 neurons. Dm8 neurons of both types are born in excess, with Dm8s that are not connected to their cognate R7s undergoing apoptosis. In the presence of extra R7s in butterfly-like retinas, additional Dm8s are retained, leading to two Dm8s per medulla column. We propose a model in which population-level variation in the ratio of R7 subtypes produced in the retina maintains an excess pool of two types of potentially interacting partners in the brain, thus providing developmental flexibility which allows brains to accommodate additional inputs. We demonstrate how excess neurons that are eliminated in normal development facilitated the expansion of color vision in butterflies, and similar cooption events may represent a general mechanism of neural evolution.

As animal brains evolve increased complexity, new neurons must integrate into established networks without disrupting preexisting neural function. Such a process must modify existing circuits, genes, or developmental mechanisms to accommodate new inputs without adversely impacting fitness^1–4^. Understanding how such changes have occurred is essential for uncovering the mechanisms that enable innovation in brains and sensory systems across species.

Neurons are often initially generated in excess during development. The neurotrophic hypothesis explains this process as a competition for limited survival signals: only neurons that form appropriate synaptic connections receive sufficient support, while others are eliminated^5,6^. Such overproduction may also facilitate evolutionary change by enabling new inputs to be matched with a flexible pool of potential partners^7^. Whether such mechanisms have been used during the integration of additional neurons during evolution has not been explored.

The insect visual system serves as a complex yet tractable model for the study of neural development and evolution. Like other insects, *Drosophila melanogaster* compound eyes are composed of individual repeating unit eyes (ommatidia). Each ommatidium contains eight photoreceptors (PRs, R1–8). *Drosophila* retinas contain two subtypes of ommatidia that are distributed randomly across the eye in a 70/30 ratio. The decision controlling which ommatidial subtype is produced is controlled by a probabilistic decision to express the transcription factor Spineless (Ss) in each R7 PR, yielding a mosaic of 70% Ss-ON and 30% Ss-OFF fates^8,9^. The Ss outcome controls which Rhodopsin (Rh) protein is expressed in R7, enabling color comparisons and color vision (reviewed in ^10^). Ommatidia with Ss-ON R7s are called “yellow” ommatidia because of the color of the R7 rhabdom in transmitted light^11^ and express Rh4 in yellow R7 (yR7) and Rh6 in the matching yellow R8 (yR8). Ommatidia with Ss-OFF R7s are called “pale” and express Rh3 in pale R7 (pR7) and Rh5 in pale R8 (pR8)^10^.

Butterflies often rely on color vision for finding mates and nectar. A key evolutionary innovation in butterfly ommatidia is that they contain nine PRs instead of the ancestral eight and two are R7 PRs^12–14^. This results in two independent, probabilistic decisions to express Ss, producing ommatidia that can be ON/OFF, OFF/OFF, and ON/ON for Ss expression. The two R7s express Rhs sensitive to Blue or UV light in three combinations in each ommatidium: B/UV (type I), UV/UV (type II), and B/B (type III), respectively^13^. This change in TF expression is responsible for novel eye patterning, providing butterflies with additional PR subtypes. These PRs have subsequently evolved to express new Rhs, thereby enabling expanded color comparisons^15^. For example, cabbage white butterflies (*Pieris rapae*) express different blue-sensitive Rh homologs in UV/B vs. B/B, while *Heliconius* butterflies differentially express a duplicate UV Rh homolog across PR subtypes^16,17^. The increase in butterfly retina complexity raises two questions: how are the additional R7s incorporated into ommatidia, and how are their neural projections integrated into preexisting circuits in the optic lobes?

## A common gene regulatory network controlling retinal development

In *Drosophila*, PR recruitment occurs sequentially: first R8, then R2/5, R3/4, R1/6, and finally R7 (reviewed in ^18^). Three signaling pathways coordinate R7 specification: EGFR is activated by Spitz from R2/5, Notch by Delta from R1/6, and the RTK Sevenless by Boss from R8^19,20^. The order of PR recruitment is highly conserved across the insects^14^, but how this process has been modified to recruit a second R7 in butterflies is unknown.

**Extended Data Fig. 1:**
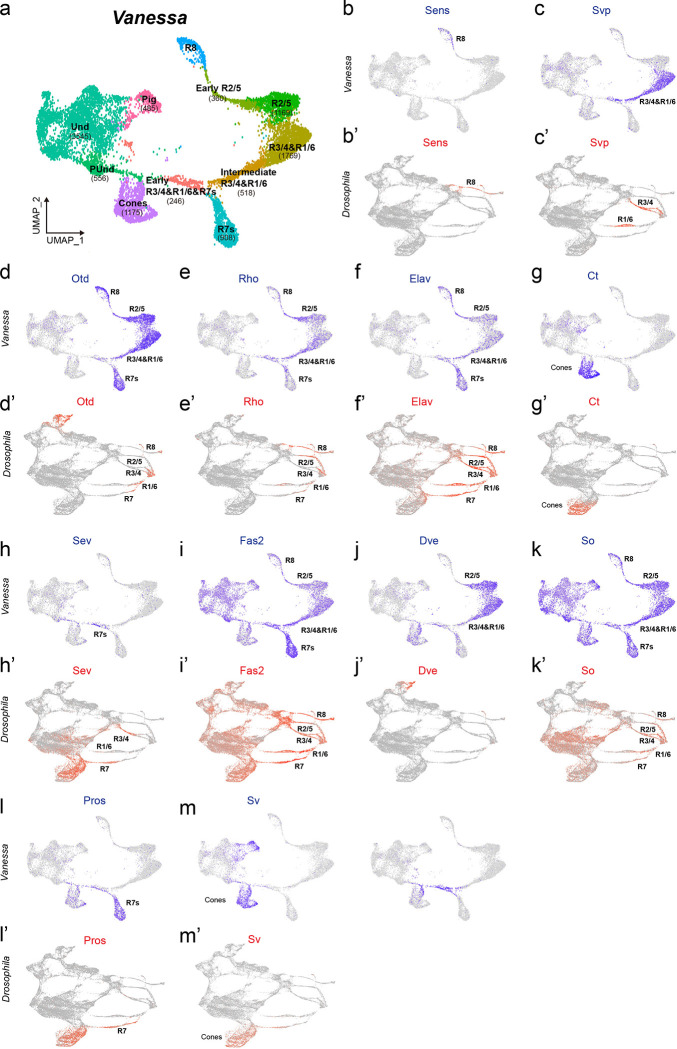
FeaturePlots highlighting TF expression in the cell types of *Drosophila* and *Vanessa.* **a**, UMAP plot showing clusters with a total of 11,106 cells from the snRNAseq data of the butterfly P20 pupal retina. The number of cells in each cluster is shown in brackets. Early R3/4/1/6 + Intermediate R3/4/1/6 = 764, Early R2/5 = 360, **b-n**, FeaturePlots showing the expression of TFs in PRs of *Drosophila* and *Vanessa*. Und, undifferentiated; PUnd, posterior undifferentiated; Pig, pigment.

**Extended Data Fig.2:**
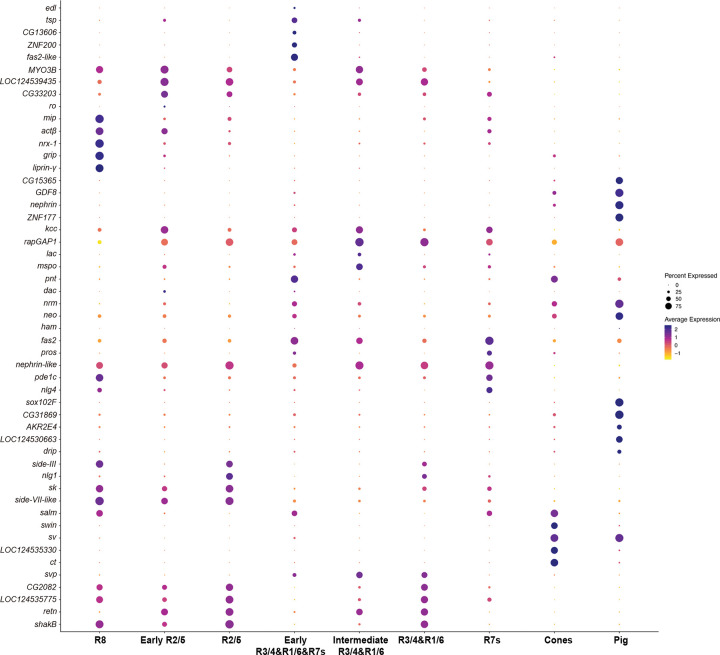
Marker gene expression within cell types. DotPlot showing the top 51 genes associated with each cell type cluster.

**Fig. 1:**
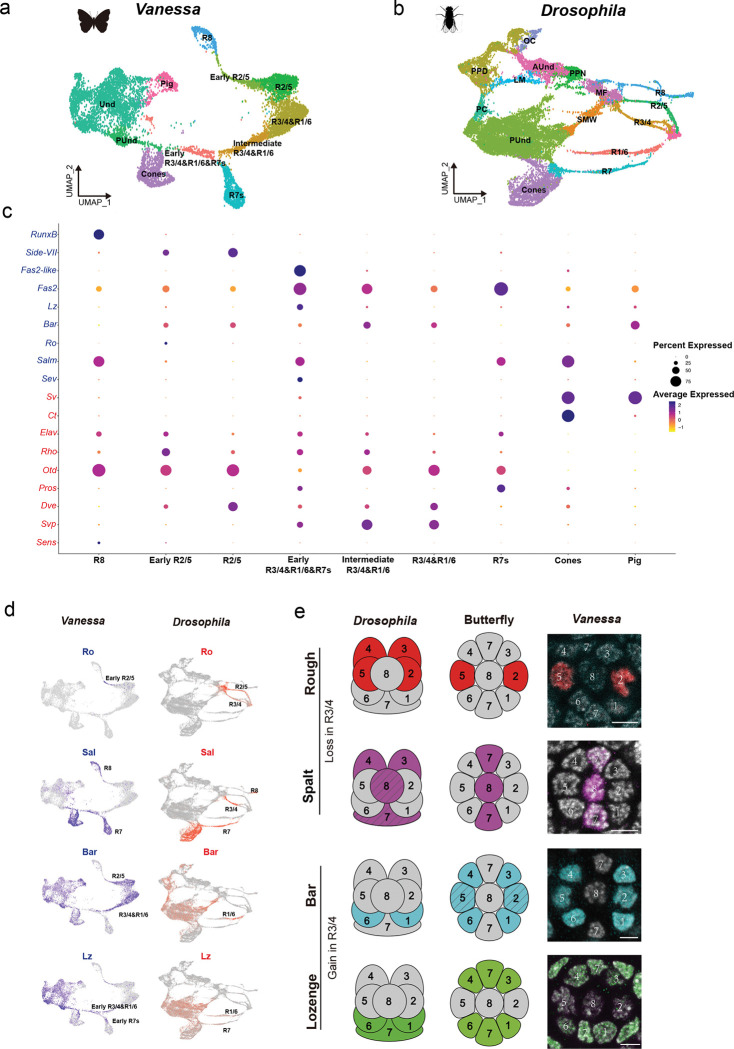
snRNAseq comparison reveals differential TF expression patterns in the developing retina between *Drosophila* and butterfly. **a,b,** UMAP plot showing PR clusters from snRNAseq data of the butterfly P20 pupal retina (**a**), and the late larval eye disc in *Drosophila* (**b**). **c**, Dot plot showing TF expression in the butterfly retina. TFs in blue are differentially expressed between *Drosophila* and butterfly, while TFs in red are deeply conserved in both species. **d**, Feature plots highlighting four key TFs expressed in PRs between *Drosophila* and butterfly. **e**, Left: schematic representation of the expression patterns of Rough (red), Spalt (magenta), Bar (cyan) and Lozenge (green) in the *Drosophila* and butterfly ommatidium. R1–6 are outer PRs; R7/8 are inner PRs. In *Drosophila*, Rough is expressed in R2/5&R3/4, but is lost from R3/4 in butterfly. Spalt is expressed in R3/4 near the MF and later in R7/8 (shown with slashes) in *Drosophila*, but is lost in R3/4 in butterfly. Bar is expressed in R1/6 in *Drosophila*, but is gained in R3/4 and later in R2/5 (slashes) in butterfly. Lozenge is expressed in R1/6 & R7 in *Drosophila*, but is gained in R3/4 in butterfly. Right: Antibody stains show the expression of Rough, Spalt, Bar and Lozenge in a single ommatidium of the pupal retina in the butterfly *V. cardui*. Scale bars, 5 μm. Und, undifferentiated; PUnd, posterior undifferentiated; Pig, pigment; OC, ocelli; PPD, anterior peripodial; PC, posterior cuboidal margin peripodium, LM, lateral margin peripodium; AUnd, anterior undifferentiated; MF, morphogenetic furrow; SMW, second mitotic wave.

To identify genes involved in PR specification and the recruitment of the extra R7s in butterfly ommatidia, we performed single nucleus RNA sequencing (snRNAseq) on developing retinas of the butterfly *Vanessa cardui* at 20% pupation (P20), capturing all cell types in the developing retina (Fig. 1a). We compared this data to previously published single cell RNA sequencing (scRNAseq) data from developing L3-stage *Drosophila* retinas (Fig. 1b)^21^. We identified the specific cell types corresponding to each cluster using combinations of markers that are well established in *Drosophila* and which are highly conserved across the insects, including Spalt (Sal) for R7 and R8, Prospero (Pros) for R7, Defective proventriculus (Dve) for “outer” PRs R1–6, and Senseless (Sens) for R8^13,14^. Additional genes that are expressed in homologous cell types between *Drosophila* and butterflies include Sevenup (Svp) in R3/4/1/6, Orthodenticle (Otd/Oc) and Elav in all PRs, Rhomboid (Rho) and Sevenless (Sev) in differentiating PRs, and Cut (Ct) and Shaven/dPax2 (Sv) in cone cells (Fig. 1c and Extended data Fig. 1&2). The conserved expression of these TFs and signaling pathway members in homologous retina cell types suggests they are components of the “insect eye ground plan”^14^.

**Extended Data Fig. 3:**
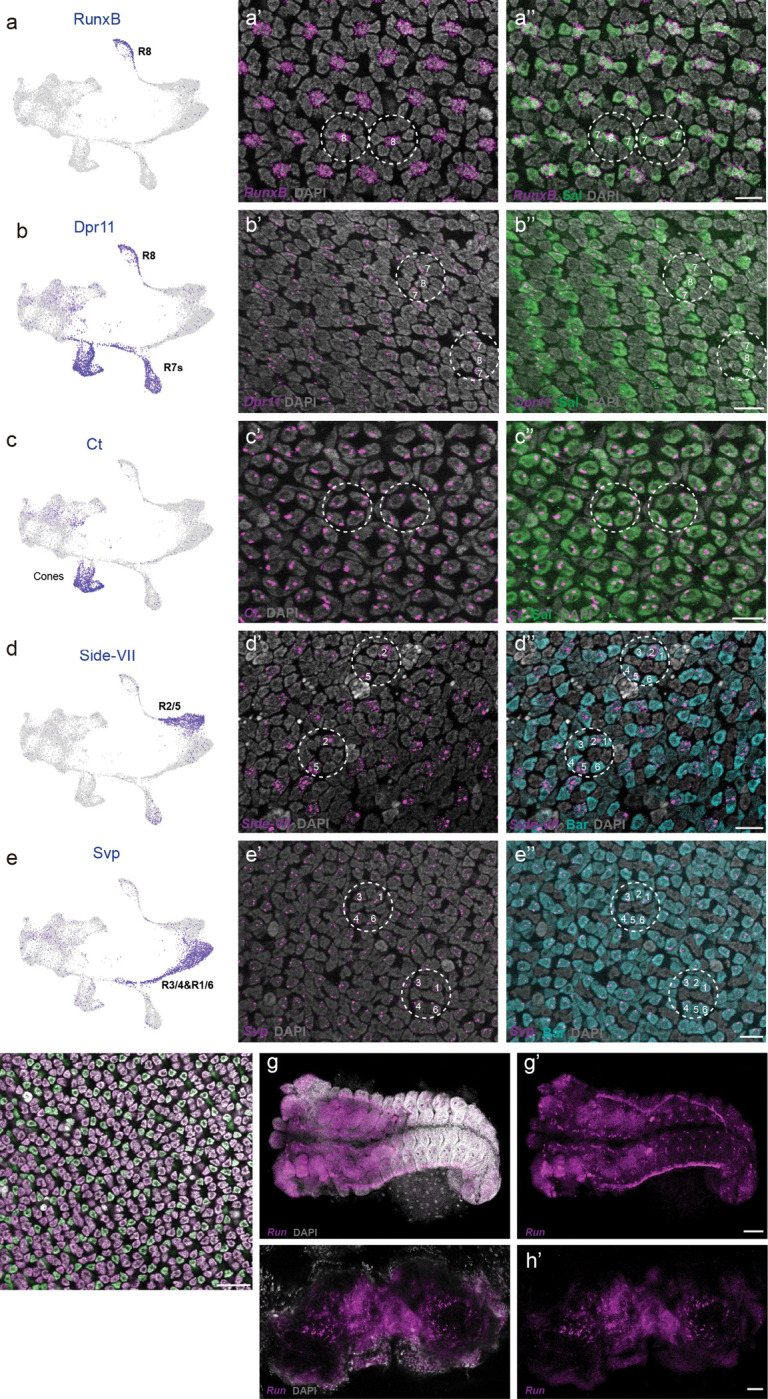
Validation of cell type clusters using HCR *in situ* hybridization and antibody staining in *Vanessa.* **a-a”**, RunxB is expressed in R8 cells. FeaturePlot showing *RunxB* expression in R8 cluster (**a**). HCR *in situ* hybridization combined with antibody staining shows *RunxB* mRNA expression (magenta) in R8 (**a’**), Sal (green) labels R8 and R7s (**a”**). **b-b”**, Dpr11 is expressed in R8 and R7 cells. FeaturePlot showing *Dpr11* expression in R8 and R7s cluster (**b**). HCR *in situ* hybridization combined with antibody staining shows *Dpr11* mRNA expression (magenta) in R8 and R7s (**b’**), Sal (green) labels R8 and R7s (**b”**). **c-c”**, Ct is expressed in cone cells. FeaturePlot showing *Ct* expression in cone cells cluster (**c**). HCR *in situ* hybridization combined with antibody staining shows *Ct* mRNA expression (magenta) in cone cells (**c’**), Sal (green) labels cone cells (**c”**). **d-d”**, Side-VII is expressed in R2/5 cells. FeaturePlot showing *Side-VII* expression in R2/5 cells cluster (**d**). HCR *in situ* hybridization combined with antibody staining shows *Side-VII* mRNA expression (magenta) in R2/5 (**d’**), Bar labels outer PRs, R1–6 (cyan) (**d”**). **e-e”**, Svp is expressed in R3/4 and R1/6 cells. FeaturePlot showing *Svp* expression in R3/4&R1/6 cells cluster (**e**). HCR *in situ* hybridization combined with antibody staining shows *Svp* mRNA expression (magenta) in R3/4 and R1/6 (**e’**), Bar (cyan) labels outer PRs, R1–6 (**e”**). **a’**-**e”**, scale bar 10μm. **f**, *Runt* mRNA expression is not detected by HCR *in situ* hybridization combined with antibody staining, only Sal (red) and Bar (cyan) expression is observed. Scale bar 20μm. **g-h’**, *Runt* mRNA expression (magenta) is observed in the embryo (**g**,**g’**) and adult brain (**h**,**h’**). **g**-**g’**, scale bar 100μm. **h**-**h’**, scale bar 50μm. All nuclei are labeled with DAPI (gray).

We also identified genes that are expressed differently between flies and butterflies (Fig. 1c and Extended Data Fig. 3). While Runt is coexpressed with Ss and UV-sensitive Rh4 in *Drosophila* R7 PRs^22^, Runt expression is lost in butterfly PRs (Extended Data Fig. 3). Runt ortholog RunxB is instead gained in butterfly R8 (Extended Data Fig. 3a). A copy of Fasciclin-2-like (Fas2-like) that is not present in *Drosophila* is expressed primarily during early PR differentiation in *Vanessa* (Extended Data Fig.1). We also observed R2/5-specific expression of Side-VII, which has not been reported in *Drosophila*. We next used HCR *in situ* hybridization to validate the expression patterns of a subset of these genes, and found that they largely match our snRNAseq results (Extended Data Fig. 3).

Strikingly, the cluster corresponding to the *Drosophila* R3/4 cell type is absent in *Vanessa*. Distinct clusters corresponding to R2/5, R3/4, and R1/6 PRs that are recruited together during development can be identified by markers in *Drosophila*^21^. In contrast, the UMAP plot for *Vanessa* shows a distinct R2/5 cluster and a combined R3/4/1/6 cluster approximately twice the size of the R2/5 cluster (Extended data Fig.1a). This relative proportion suggests cells in the R3/4 position instead differentiate as R1/6-type in the butterfly.

**Extended Data Fig. 4:**
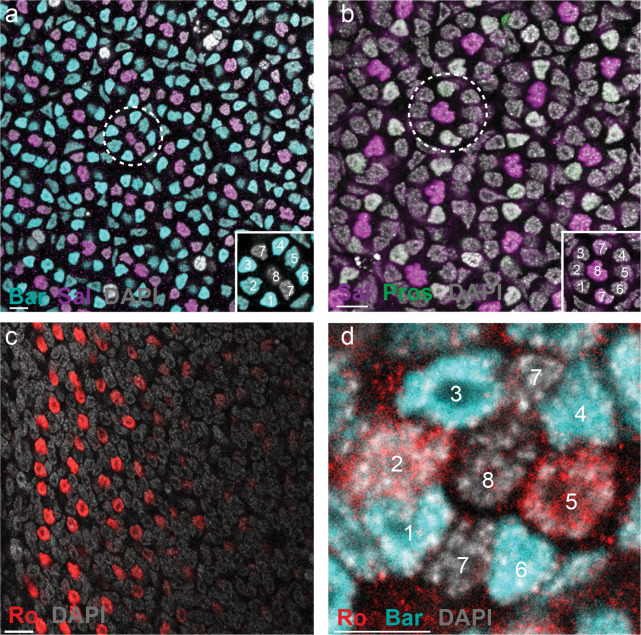
Expression of four TFs in butterfly PRs. **a**, Antibody stains showing ommatidia in the butterfly *Vanessa*. Bar (cyan) is expressed in outer PRs, R1–6. An ommatidium with nine PRs is shown within a dashed circle (also see inset). Scale bar, 10μm. **b**, Sal (magenta) is expressed in R8 and R7 PRs. Pros (green) is expressed in two R7s. The dashed circle highlights an ommatidium (also see inset). Scale bar, 10μm. **c**, Ro (red) is transiently expressed near the morphogenetic furrow. Scale bar, 10μm. **d**, An ommatidium near morphogenetic furrow showing Ro expression in R2/5, and Bar expression in R3/4 and R1/6. Scale bar, 5μm. All nuclei are labeled with DAPI (gray).

To determine whether cells in the R3/4 position gain R1/6-like gene expression, we identified genes which are differentially expressed in these pairs in *Drosophila* and evaluated their expression in *Vanessa* using immunohistochemistry (Fig.1). In *Drosophila*, R3/4 are defined by their expression of the TFs Rough (Ro) and Sal, while R1/6 instead express Lozenge (Lz) and BarH1 (Fig.1). To evaluate expression in *Vanessa*, we used an existing cross-reactive antibody for Sal and generated new butterfly-specific antibodies for Ro, Lz, and Bar homologs (see Methods). In *Vanessa*, cells in the R3/4 position adopt R1/6-like fate (Fig.1e and Extended Data Fig. 4), expressing Lz and Bar but not Ro or Sal (Extended Data Fig. 4a–c), resulting in symmetric patterns of expression in each ommatidium. This confirms that cells in the R3/4 position adopt R1/6-like identity in butterflies.

The conversion of R3/4 cells to R1/6 identity suggests an intriguing hypothesis: that cells in the R3/4 position in butterflies turn on the developmental program normally used by R1/6 to recruit a neighboring cell to become R7. In *Drosophila*, the R3/4 pair differ from R1/6 in that once the former are incorporated into the ommatidium, they adhere strongly and prevent cells between them from gaining contact with the central R8^23,24^. R1/6, however, remain separate from each other allowing another cell (the R7 precursor) to lie between them and contact R8. In developing ommatidia, a cell initially lies between R3/4s but is excluded from R8 as R3/4 zip up together. This is the mystery cell (M cell), which subsequently regresses from the cluster and rejoins the surrounding cell pool^23,24^. We propose that the M cell is the precursor of the supernumerary R7 in the butterfly, and the respecification of R3/4 as R1/6 prevents them from zipping up, allowing the cell to gain contact with R8 and differentiate as an R7 PR (diagram in Fig. 4a).

We set out to determine which specific changes in gene expression are sufficient for the conversion in R3/4 to R1/6 fate, and whether that conversion is sufficient to allow recruitment of a second, butterfly-like R7 in the position of the mystery cell, opposite the existing R7.

## Lz expression transforms R3/4 into R1/6 and allows the inclusion of the supernumerary R7

The top differentially expressed candidate TFs between R1/6 and R3/4 cell types were Ro, Lz, Sal, and Bar. While some regulatory relationships between TFs had been examined previously^25–30^, earlier studies did not focus on whether changes in R3/4 fate can induce recruitment of an adjacent R7, and this became our objective. We first conducted gene loss-of-function experiments and examined the expression of Lz and Bar as markers of R1/6 fate, and Sal and Ro as markers of R3/4 fate.

**Fig. 2:**
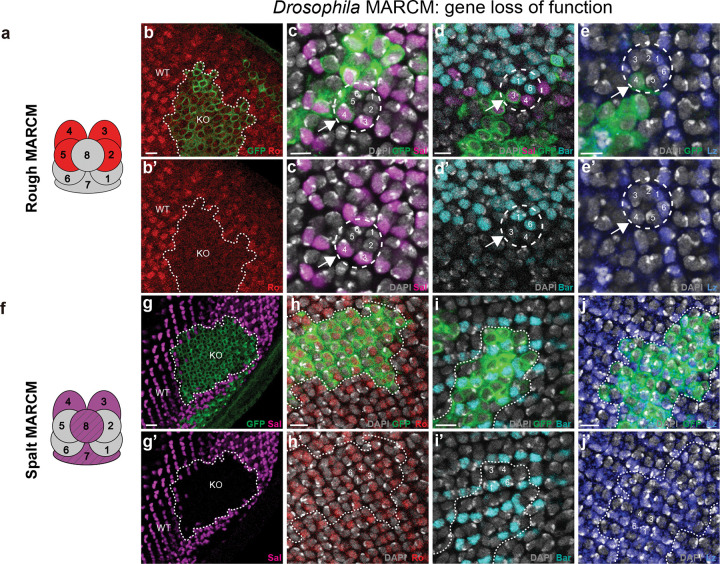
Loss of function of Rough and Spalt in third-instar larval eye disc of *Drosophila*. **a**, Schematic showing Rough expression in R2/5, R3/4 in *Drosophila*. **b,b’**, Rough (red) is lost in MARCM mutant clones (KO regions labelled by GFP, green), as compared to wild type (WT) region. **c-e’,** loss of Rough does not affect Spalt, Bar and Lozenge expression. The circle in (**c,c’**) highlights an ommatidium where Rough (GFP, green) is lost in R4 (arrows), while Spalt expression in R4 (magenta) is unchanged. The circle in (**d,d’**) highlights an ommatidium where Rough is lost in R3 (arrows), and Bar is not gained in R3/4 (Spalt-positive cells in magenta), and normally expressed in R1/6 (cyan). The circle in (**e,e’**) highlights an ommatidium where Rough is lost in R4 (arrows), Lozenge is not gained in R3/4, and normally expressed in R1/6 (blue). **f**, schematic showing Spalt expression in R3/4, R7/8 in *Drosophila*. **g-g’**, Spalt (magenta) is lost in MARCM mutant clones (KO regions labelled by GFP, green), compare to WT region. **h-j’**. loss of Spalt does not affect Rough (red in **h,h’**), Bar (cyan in **i,i’**), and Lozenge (blue in **j,j’**). An ommatidium labelled with numbers in white is shown in the Spalt MARCM KO regions.

We made MARCM clones in *Drosophila* eye discs using null alleles of *ro* and *sal* (Fig. 2). In Ro loss-of-function clones (marked by GFP in Fig. 2b–e), GFP-marked cells in the R3/4 position do not lose Sal expression (Fig. 2c) and do not gain Bar (Fig. 2d) or Lz expression (Fig. 2e). This indicates that Ro expression in R3/4 is not required for Sal activation or for repression of Bar or Lz. In Sal loss-of-function clones (Fig. 2g–j), GFP-marked cells in the R3/4 position do not lose Ro (Fig. 2h) or gain Bar (Fig. 2i) or Lz (Fig. 2j), indicating that Sal is not required for Ro activation, or for repression of Bar or Lz. It was previously observed that 4.8% of R3/4 cells in Sal mutant clones are Bar-positive^31^. While this change is butterfly-like, it is rare enough to suggest that neither Ro nor Sal are fully upstream of each other, Bar, or Lz, and their removal is not sufficient for recruitment of a neighboring R7.

**Extended Data Fig. 5:**
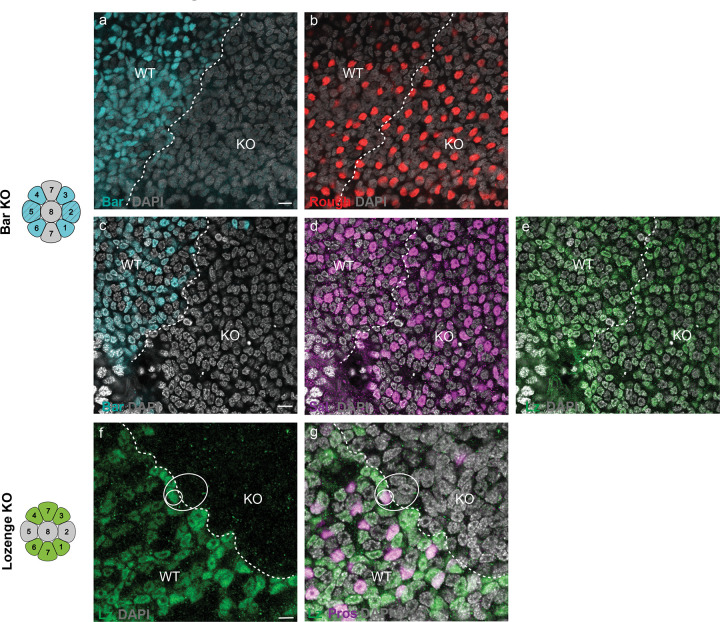
The effects of Bar and Lozenge loss-of-function in the butterfly *Vanessa* retina. **a-e**, CRISPR-induced mosaic knockout of *Bar* does not affect the expression of Ro (red, **b**), Sal (magenta, **d**) or Lz (green, **e**) in the pupal retina of *Vanessa*. Bar (cyan, **a**,**c**) is lost in KO regions compared to the WT region. The boundary between WT and Bar KO regions is indicated by a white dashed line. Scale bar, 5μm. **f-g**, CRISPR-induced mosaic knockout of *lz* results in the loss of R7s in the pupal retina of *Vanessa*. The solid white circle highlights an ommatidium near the clone boundary that has lost one R7 (the remaining R7 is stained with Lz (green, **f**) and Pros (magenta, **g**) on WT side) indicating that Lz is directly required in R7 recruitment. All nuclei are labeled with DAPI (gray). Scale bar, 10μm.

**Fig. 3:**
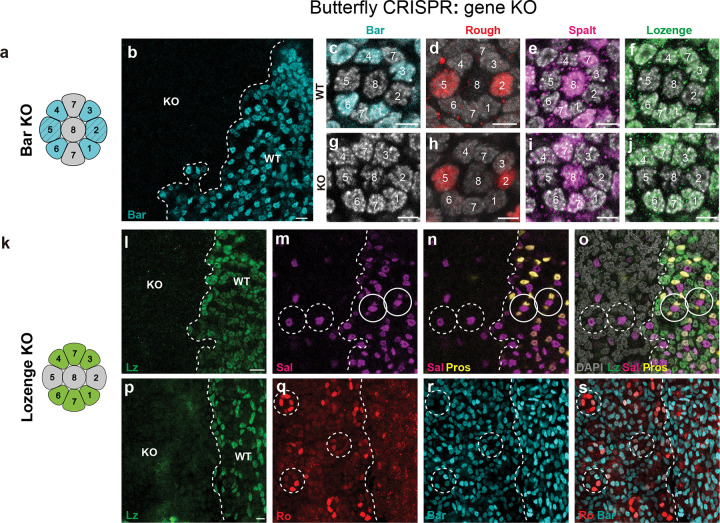
Lozenge is necessary for the recruitment of two R7s in the butterfly. **a**, Schematic showing Bar expression in R1–6 in a butterfly ommatidium. **b-j**, CRISPR mosaic knockout of Bar does not affect the expression of Rough, Spalt or Lozenge in the pupal retina of *V. cardui*. **b**, Bar is lost in KO regions compared to WT region (cyan). The boundary between WT and Bar KO regions is indicated by a white dashed line. **c-f,** a single ommatidium in the WT region. **g,** a single ommatidium in the Bar KO region, where Rough (red, **h**), Spalt (magenta, **i**) and Lozenge (green, **j**) are normally expressed. **k**, schematic showing Lozenge expression in R1/6, R3/4, and R7s in butterfly. **i-o**, CRISPR mosaic knockouts of Lozenge result in the loss of R7s. Dashed circles highlight example ommatidia in Lozenge KO region, as compared to solid circles in WT regions. The boundary between WT and lozenge KO regions is indicated by a white dashed line. In the WT region, Spalt (magenta) is expressed in R8 and two R7s per ommatidium, while Prospero (yellow) is expressed in two R7s. In KO regions, Prospero and Spalt coexpressing R7 cells are lost, but not Spalt-only expression cells (R8s). **p-s,** In Lozenge KO regions, we observe a variable number of outer PRs (dashed circles), indicating that R7s can be variably lost or transformed into outer PRs. Outer PRs within KO regions show mixed identity, with variable levels of Rough (red in **q**) and Bar (cyan in **r**) expression, suggesting derepression of Rough in the absence of Lozenge.

We then used CRISPR/Cas9 in *Vanessa* to knock out *bar* and *lz*, producing mosaic mutant animals. We used immunohistochemistry to identify regions of tissue where the targeted protein is lost. In Bar loss-of-function clones, Ro, Sal, and Lz expression is unaffected (Fig. 3c–j and Extended Data Fig. 5a–e). In Lz loss-of-function clones, however, both R7 PRs are lost as indicated by loss of Pros and Sal expressing cells flanking the Sal-positive R8 (Fig. 3l–o). When examining the boundaries of Lz knockout clones, we observed examples of ommatidia where Lz positive outer PRs (R1/6 type) are adjacent to a Lz-negative clone and have a missing R7, indicating that Lz expression is also required autonomously in R7 (Extended Data Fig. 5f,g), (as in *Drosophila*)^27^. Within null clones, the number of PRs around each R8 is variable from 6 to 8, suggesting that R7s can either be variably lost or transformed into outer PRs, as observed in *Drosophila lz* mutant ommatidia^25,26^. Outer PRs within Lz mutant regions have variable levels of Bar and Ro expression (Fig. 3p–s), suggesting a derepression of Ro in the absence of Lz. In general, we observed an inverse relationship between Ro and Bar expression, suggesting that Ro represses Bar in *Vanessa* (Fig. 3q–s). Overall, these results demonstrate that Lz is necessary for the recruitment of both R7s, and suggest that Ro, Bar, and Sal are downstream of Lz expression in defining R1/6 fate.

Loss-of-function experiments suggested that Lz is a key player in the conversion of R3/4 to R1/6-type fate. We next sought to add Lz to R3/4 cells in *Drosophila,* to determine whether this change is sufficient to induce recruitment of a neighboring R7. A similar experiment had been performed previously with an unexpected result: R3 and R4 themselves were variably transformed into R7^32^, and it was determined that Notch levels are important in whether they are converted. R1/6 express Lz and have low Notch (N) activity, while R3/4 lack Lz and have high N signaling. N signaling in R3/4 is critical for coordinated ommatidial rotation in *Drosophila,* but potentially less important in butterflies, which have fused rhabdoms and lack neural superposition^33^. The goal became to ectopically express Lz in R3/4 while simultaneously reducing N activity to determine whether cells in the R3/4 position would become R1/6-like and allow the incorporation of the M cell as an R7 PR.

**Fig. 4:**
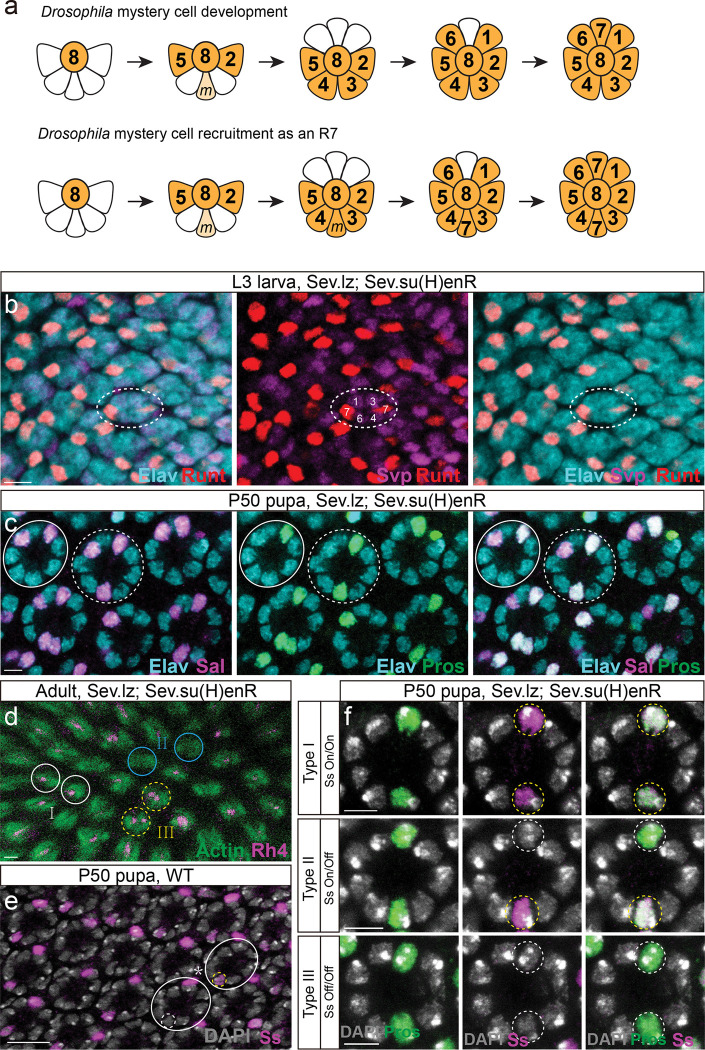
Conversion of R3/4 toward R1/6 fate is sufficient to create butterfly-like ommatidia in *Drosophila*. **a**, Schematics show the order of PR recruitment in *Drosophila* eye imaginal disc. The mystery cell(s) do not present after R2/5 differentiation in WT flies (top), but the mystery cell(s) recruit as R7s in flies with the genotype sev.lz, sev.su(H)enR flies (bottom). **b,** Third-instar larval eye disc with genotype sev.lz, sev.su(H)enR. Ommatidia (Elav labeling PRs in cyan) are observed with two R7s (Runt-positive, red), while R3/4&R1/6 are labelled by Svp (magenta). **c**, P50 retina with genotype sev.lz, sev.su(H)enR. Ommatidia are observed with two R7s (Spalt in magenta and Prospero in green; coexpression in white; Elav in cyan). A solid circle shows a WT ommatidium, while a dotted circle highlights an ommatidium with an additional R7. **d**, Adult retina of sev.lz, sev.su(H)enR. Ommatidia with two R7s show either +/+ (III, yellow dashed circles), +/− (I, white solid circles), or −/− (II, blue solid circles) for Rh4. **e**, In the P50 WT retina, two types of ommatidia, circled in solid white, are observed: Ss^on^ (dotted circle in yellow) and Ss^off^ (dotted circle in white). **f**, In P50 retina of sev.lz, sev.su(H)enR, three types of ommatidia are observed: Ss^on^/ Ss^on^ (type I), Ss^on^/ Ss^off^ (type II) and Ss^off^ / Ss^off^ (type III).

We tested a number of drivers (see Extended Data) and found that supplying Lz to R3/4 using the construct sev.lz^32^ while reducing N activity using the construct sev.Su(H)EnR^19^ produces butterfly-like ommatidia. We identified ommatidia near the MF at L3 that have nine PRs with two R7s in the same position as in butterflies, in the position of the M cell (Fig.4b). This fate becomes especially clear at pupal stages (Fig.4c). This phenotype was not fully penetrant, with some *Drosophila*-like ommatidia remaining, and rare ommatidia recruit two extra R7s at the position of the mystery cell (Fig.4c). A variable number of mystery cells labeled M1 and M2 had been previously reported^24^. While others have reported the conversion of R1/6 directly into R7^32^, or the recruitment of M as an additional outer PR (e.g. ^34,35^, our experiment was the first to successfully recruit M as an R7 and produce butterfly-like ommatidia.

By pupal stages (P50), these butterfly-like ommatidia make a stochastic, cell intrinsic choice to express Ss: we observed ommatidia with either two, one, or no Ss-positive R7s (Fig. 4f, dashed circles), in contrast to wild type that have ommatidia that are either Ss-ON or Ss-OFF in the single R7 (Fig. 4e, dashed circles). Finally, we examined Rh expression downstream of Ss in adult retinas and found ommatidia that have two R7s which express Rh4 in one, two or no R7, showing the presence of additional color-sensitive R7 PRs (Fig. 4d). Therefore, when the mystery cell is recruited as an additional R7 PR, it leads to three stochastically distributed ommatidial types instead of two. Together, these results demonstrate that conversion of R3/4 toward R1/6 fate is sufficient to implement the developmental program used to recruit a neighboring R7 PR.

## How does the optic lobe accommodate two R7 cells?

We next sought to understand how input from additional R7 PRs could be accommodated at the level of the optic lobe, the part of the fly brain that processes visual information. Were additional, complementary genetic changes required to integrate the additional R7s into color vision circuits in the brain?

In *Drosophila*, each R7 and R8 projects to a corresponding column in the medulla where they connect to specific cell types. The primary interacting partner of R7 PRs is Dm8^36^. Although all Dm8s interact with multiple R7s, they establish preferential synapses with a single R7 as their “home column”. Thinner lateral projections to neighboring medulla columns are thought to enable center-surround comparisons^37,38^.

Recent work identified two types of Dm8s that interact with stochastically specified R7s called “yellow” or “pale” Dm8s (yDm8 or pDm8), which interact with Ss-ON yR7s or Ss-OFF pR7s, respectively^37,38^. The two Dm8 subtypes are specified deterministically in different regions and send projections to find partner R7s of the right type^37,38^. yR7s express a cell surface protein Dpr11 which interacts with DIPγ expressed in yDm8 cells^39^. This interaction allows recognition of yR7 with its cognate yDm8 and provides a survival signal: Dm8s that do not find connections undergoing apoptosis^37,38^. A similar matching mechanism must exist for the pR7 and pDm8, though the specific proteins that mediate pDm8 survival remain unidentified. This matching process ensures that cell types produced via a stochastic decision in the retina are met with the appropriate types of target cells that are specified independently and deterministically.

Both types of Dm8s are produced in an excess of ~30%^37,38^. We propose that this surplus allows the system to accommodate population-level variation in the stochastic ratio of R7 subtypes. While the population averages 70% Ss ON R7s, the stochastic ratio in the retina was shown to range from 19% to 83% Ss-ON in inbred lines^40^, demonstrating significant potential for variability. In this scenario, individuals that inherit higher or lower R7 subtype ratios produce enough excess Dm8s of each type to accommodate variation in the stochastic ratio. Such variation could provide a reason for selection to maintain an excess pool of potentially interacting Dm8s of each type.

We hypothesize that an evolutionary change in the retina that increases the number of R7s could be immediately accommodated by the extra Dm8s. To test this model, we assessed whether butterflies have two Dm8 home column projections in each medulla column, and then whether *Drosophila* modified to produce additional R7s can accommodate these additional R7s by adding additional Dm8s.

**Fig. 5:**
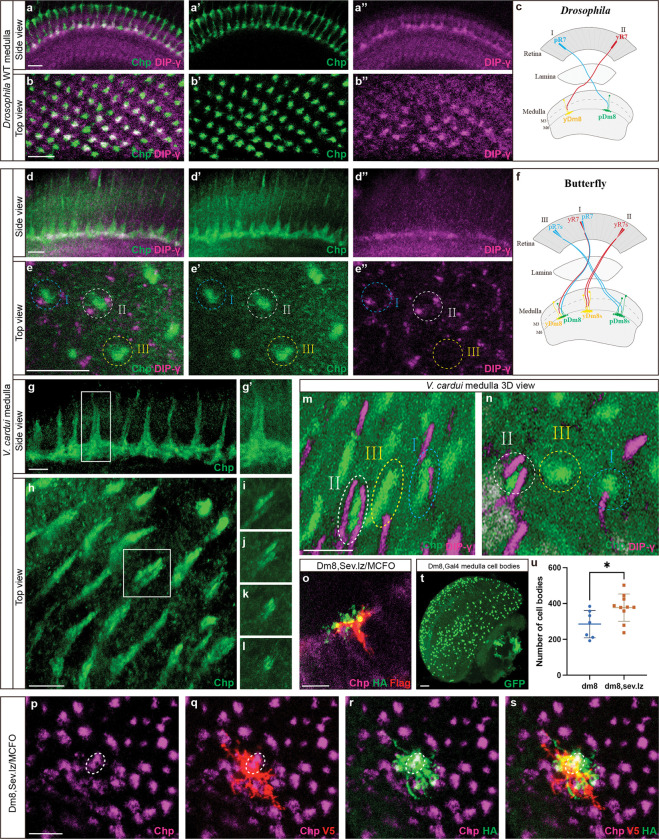
Additional R7 PRs are accommodated at the level of the brain. **a**, Sideview of the P50 medulla in *Drosophila*. R7 axons are labelled by Chaoptin (green), and Dip-γ (magenta) marks yDm8s. **b**, Top-down view of the P50 medulla in *Drosophila*, R7 terminals are labelled by chaoptin (green), and yDm8 are marked by Dip-γ (magenta). **c**, Schematic depicting the interaction of yDm8s with Ss-ON yR7s, while pDm8s interact with Ss-OFF pR7s in *Drosophila*. **d**, Sideview of the adult butterfly medulla. R7 axons are labelled by chaoptin (green), and Dm8s are labelled by Dip-γ (magenta). **e**,Top-down view of the adult butterfly medulla, DIPγ signal is observed alongside paired R7 terminals: present on one side (I, blue dashed circle), both sides (II, white dashed circle), or neither side (III, yellow dashed circle). **f**, Schematic showing three types of interaction between R7s and Dm8s in the butterfly. **g-i**, Two R7 axons within single columns in butterfly medulla. **m,n**, 3D views show DIPγ-positive projections along both sides of some R7 terminal pairs: one side (I, blue dashed circle), both sides (II, white dashed circle), or neither side (III, yellow dashed circle). **o-s**, MCFO labelling of two Dm8s sharing a single “home column” in sev.lz flies, which produces extra R7s per ommatidium. Two Dm8s (labeling with V5 and HA, red and green) connect to two R7 terminal axons (Chp, magenta) in one column. **t**, Dm8 cell bodies are labelled with GFP in WT. **u**, Quantification of Dm8 number in WT vs. sev.lz adult medulla (WT, n=7, sev.lz, n=10). A significant difference is indicated by an asterisk (Student’s *t*-test, *P*<0.05).

Fig. 5a shows a *Drosophila* medulla, with R7 and R8 PR terminals marked by Chaoptin (Chp). R7 axons terminate in the deeper M6 layer and interact with yDm8 projections which are labeled by DIPγ expression, as shown previously, as well as with unlabeled pDm8s^37,38^ (Fig. 5a,b). Corresponding side views of a butterfly medulla show similar co-localization of Chp and DIPγ signal in a single medulla layer. Unlike in *Drosophila*, we observed two distinct R7 axons within single medulla columns in butterflies (Fig. 5g, h–l). DIPγ signal can be seen alongside the paired R7 terminals in a variable fashion: sometimes present on one side, both sides, or neither side, indicating one, two, or no yDm8s (Fig. 5e,m,n). There is no marker for pDm8s or a general marker for all Dm8s in butterflies, but the presence of two DIPγ-positive projections in single columns suggests butterflies have two Dm8s per medulla column.

We next evaluated the effect of R7 loss on Dm8 number in butterfly medulla columns. We showed previously that Lz KO causes loss of R7 PRs (Fig. 3k–o). We examined the optic lobes of Lz CRISPR mosaic somatic mutant butterflies and observed significant gaps in DIPγ expression in regions where R7 terminals are missing (Extended Data Fig. 8), indicating that R7 terminals are required for the retention of DIPγ-positive Dm8 neurons in retinotopic positions. This provides evidence that, as in the fly, a trophic signal from yR7s is also required for yDm8 survival in butterflies, and that DIPγ plays a conserved role in yR7/yDm8 patterning downstream of the stochastic choice in the retina.

We next examined how a higher R7 number impacts Dm8 specification in *Drosophila*. We used MCFO sparse labeling^41^ to label a small number of Dm8 neurons per medulla in both WT and the sev.lz background^32^, which produces extra R7s per ommatidium (Fig. 5o–s). We identified cases where two Dm8s shared a single “home column” which we did not observe in WT controls and which has not been observed in previous studies of Dm8 morphology with light microscopy^37,38^ or electron microscopy^42,43^. In the two cases shown, although the labeled Dm8s have the same home column (Fig. 5o), their lateral projections to neighboring columns differ, suggesting different center-surround connections. These results suggest that two Dm8s can occupy the same home column when additional R7s are present per ommatidium in the retina.

Finally, we found that the overall number of Dm8 neurons retained through development increases when additional R7s are recruited in the fly retina. We quantified the number of Dm8s present in WT vs sev.lz adult brains (Fig. 5t) and observed a 32% increase per brain when additional R7s are present (Fig. 5u, Welch’s *t* test, df=15, *P*=0.027). Together, this evidence suggests butterflies produce two Dm8 neurons per medulla column and that *Drosophila* can position two Dm8s per medulla column when additional R7s project to this column. This suggests that some of the previously excess Dm8s are instead used to form new connections.

## Discussion

The developmental mechanisms that control core insect eye development are deeply conserved, despite morphological differences and divergence in visual function across the insects^14^. There is evidence that the same signaling pathways are used in this patterning process and that the TFs that define PR types are conserved in species as distant as crickets^14^. The canonical eight-PR, single R7 arrangement is also highly conserved^14,44^. One of the most dramatic deviations from this conserved patterning system is found in the Lepidoptera: butterflies have increased the complexity of their stochastic retinal mosaics by producing two R7-type PRs, expanding their color vision^12–14^. Here, we identify the genetic and developmental basis of how butterflies produce two R7 PRs per ommatidium.

The most immediate benefit to having a second R7 per ommatidium should be an increase in both sensitivity and color resolution. UV/UV ommatidia have two UV-sensitive PRs that view the same point of the visual field, thus increasing UV sensitivity. Similarly, Blue/Blue ommatidia would have higher sensitivity to blue light. The UV/Blue ommatidial type provides the ability to discriminate both UV and Blue light in a single position, effectively increasing color resolution compared to detecting a single wavelength range in that position of the visual field. A subtler potential benefit is that while Dm8s have a central home column in the medulla that interacts with a primary R7, they also connect to a stochastic subset of neighboring ommatidial columns, enabling “center surround” comparisons. Having two R7s that each connect to a different Dm8 neuron, each with a different set of center-surround connections, could also affect color discrimination and color processing. Evolutionarily, the presence of three ommatidial types provided a place to express newly duplicated Rhs that in some cases acquired novel spectral sensitivities, such as in *Heliconius*^17^ and *Pieris*^16^. By producing additional ommatidial types, other coordinated changes could also occur, such as the evolution of ommatidia type-specific expression of green- or red-sensitive Rhs in outer PR homologs in *Papilio* swallowtails^15^ downstream of the stochastic choices in R7^13^.

We provide evidence that insect brains are able to immediately accommodate changes in the number of R7s per ommatidium. However, there are only 30% excess of each type of Dm8 produced^37,38^, leading to a potential shortage if the entire retina were modified to have two R7s per ommatidium at once. Such a change could have initially evolved in a subregion of the retina. Supporting this idea, we identified a moth species that does just this: in *Manduca sexta* hawkmoths only the ventral retina contains ommatidia with two R7s and is butterfly-like, while ommatidia in the dorsal retina have single R7s and are fly-like^14^. A later expansion in the number of Dm8s produced could enable expansion of the region containing two R7s to the full retina, as found in butterflies.

Other studies have experimentally recapitulated events during the evolution of color vision. Some primates such as squirrel monkeys are dichromatic and lack red color vision, as do other mammals including mice. Two studies introduced human red Opsin regulatory and coding sequences into PRs in either squirrel monkeys or mice and found that, amazingly, this conferred the ability to see red^3,45^. The exact mechanism and molecules that mediate this gain in ability are not fully understood. Perhaps the expansion to trichromacy in vertebrate color vision also made use of excess neurons that are normally removed during development.

The production of more neurons than are needed is common in neural development. In some cases, the excess neurons are thought to enable neural plasticity via neural activity-dependent reinforcement of a subset^7^. The example we describe in butterfly visual systems is different: excess Dm8 neurons are produced to enable matching between a stochastically patterned retina and a deterministically patterned optic lobe, with the excess produced able to accommodate population-level variation in the stochastic ratio. This mechanism results in developmental flexibility that offers an alternative to classic neurotrophic models by emphasizing not just cell number, but the need to match cell identity, providing a flexible framework for accommodating novel inputs. Whether excess neurons are used to accommodate stochastic patterning or to enable neural plasticity, their presence could provide potential for cooption into new roles during neural evolution.

## Methods

### Animals

Painted lady butterflies *Vanessa cardui* were obtained from Carolina Biological Supply Company, Burlington NC, USA. Larvae were fed on artificial diet individually in plastic cups. Adults were placed in a mesh cage with sugar water under artificial lights. Eggs were collected using sunflower stems. *Drosophila melanogaster* were reared on standard medium, and maintained in incubators at 25 °C.

### snRNAseq analysis

We dissected 16 pupal retinas at Day 2 from the butterfly *V. cardui*. After dissection, retinas were flash frozen in liquid nitrogen and stored at −80°C. We used 10x Multiome kit for library preparation with a target recovery of 6000 cells. Retinas were split into two biological replicates, each with a target sequencing depth of ~300 million reads. Sample processing and sequencing were performed at the Center for Epigenomic, University of California, San Diego. snRNAseq data were processed using the Cell Ranger pipeline (v9.0.1) with the *V. cardui* reference genome ilVanCard2.1. The combined raw matrix from Cell Ranger output had 13907 cells and a total of 577 million reads. We performed downstream analyses in R (v4.4.2) using Seurat (v5.2.1). We first filtered out low quality cells, and normalized the filtered gene expression matrix using the NormalizeData function with the LogNormalize method and a scaling factor of 10000. To visualize cell clusters using UMAP, we performed dimensionality reduction with RunPCA (npcs=150), followed by clustering and visualization with RunUMAP (dims=1:50), FindNeighbors, and FindClusters (resolution=1.0) in Seurat. Differentially expressed genes for clusters were identified using the FindAllMarkers function with a minimum expression threshold (min.pct=0.05) and a log-fold change threshold (logfc.threshold=0.05). For comparison, snRNAseq data from *Drosophila* larval eye disc were used from GEO (accession no. GSE235110)^21^.

### Fly genetics

The following genotypes of *D. melanogaster* were used: **a**) Mosaic analysis with a repressible cell marker (MARCM) clones: yw122, UAS-CD8gfp;; tubgal4, frt82, tubgal80/Tm6b (BDSC #86311), yw, hsflp; sp/Cyo; rox63, FRT82/Tm2 (BDSC #6335), UAS-CD8gfp, hsflp122; frt40a, tubgal80; tubgal4/Tm6b (BDSC #44406), yw, frt40, Df(1)sal/CyO (from Claude Desplan) **b**) MultiColor FlpOut (MCFO): pBPhsFlp2:: PEST in attP3;; HA_V5_FLAG (BDSC #64093), pBPhsFlp2:: PEST in attP3;; HA_V5_FLAG_OLLAS (BDSC #64091), w;; Dm8gal4 (BDSC #49087), w;; Dm8gal4, sev.lz/Tm6b (sev.lz from Andrew Tomlinson), yw; sp/CyO; UAS-CD8GFP/Tm6b (BDSC #7465) **c**) Mystery cells: yw,hsflp;sp/CyO; sev.lz/Tm2, yw,hsflp;;sev.Su(H)EnR/Tm2 (sev.Su(H)EnR from Andrew Tomlinson).

To generate MARCM clones, fly larvae were heat-shocked at 37 °C in a water bath for 30–45 min after a 48h egg laying period^46^. To label Dm8 neurons using MCFO, newly hatched fly adults were heat-shocked at 37 °C in a water bath for 12–20 min^47^.

### Butterfly CRISPR/Cas9 knock-out

To determine the functional roles of Bar and Lozenge in the recruitment of two R7s, we knocked out *bar* and *lozenge* in *V. cardui* using CRISPR/Cas9. The details of butterfly embryo injection were described in ^13^, and are summarized here briefly: eggs were collected 1–7h after egg laying, two sgRNA and Cas9 were co-injected with yellow sgRNA for targeting *yellow* color mutation. After injection, the hatched larvae were immediately transferred to the artificial diet until pupation. A mixture of final concentration of sgRNA/Cas9 at 250 ng/ul was used for injection. Synthetic sgRNAs were obtained from Synthego, and sequences can be found in Supplementary Table 2. Genomic DNA was extracted from the mosaic crispants and WT butterflies using the DNeasy Blood & Tissue kit (Qiagen, Germany). PCR amplification was performed using the primers listed in Supplementary Table 1, and the PCR products were purified using the FastPure Gel DNA Extraction Mini Kit (Vazyme, China). Sanger sequencing of the sgRNA-targeted regions was conducted at Azenta Life Sciences (USA). CRISPR-induced indels (*bar* and *lozenge*) were confirmed using Synthego ICE (Extended Data Fig. 9).

### Immunohistochemistry and HCR *in situ* Hybridization

Larval eye/antennal imaginal disc, pupal and adult retina and optic lobes were dissected and stained as described in detail in ^13^. Commercial primary antibodies were used as follows: sheep anti-GFP (Bio-rad 4745–1051, 1:500), mouse anti-Rough (DSHB 528456, 1:10), mouse anti-svp (DSHB 2618080, 1:100), rat anti-Elav (DSHB 528217, 1:50), mouse anti-lozenge (DSHB 528346, 1:10), mouse anti-prospero (DSHB 528440, 1:10), mouse anti-chaoptin (DSHB 528161, 1:50), rabbit anti-HA (Invitrogen 26183, 1:400); sheep anti-HA (Invitrogen OSH00021W, 1:400), chicken anti-FLAG (Exalpha Biologicals 15242, 1:1000), mouse anti-V5 (Invitrogen R960–25, 1:100), rabbit anti-V5 (Invitrogen MA5–32053, 1:400). The following antibodies were gifts: guinea pig anti-SalM (1: 400), rat anti-VcBar (1:500), guinea pig anti-Ss (1:500) guinea pig anti-runt (1:1000), guinea pig anti-rh4 (1:500) (Claude Desplan, NYU). The following antibodies were generated for this study by Genscript (Piscataway, NJ), with sequences used for antibody production listed in Supplementary Table 2: guinea pig anti-VcDIP-γ (1:200), rabbit anti-SalC (1:100), rabbit anti-VcRo (1:100), guinea pig anti-VcLz (1:100), rabbit anti-VcChaoptin (1:50). AlexaFlour secondary antibodies were used as follows: donkey anti-rabbit-405 (1:500), donkey anti-rabbit-488 (1:500), donkey anti-mouse-488 (1:500), donkey anti-rat-488 (1:500), donkey anti-sheep-488 (1:500), donkey anti-mouse-555 (1:500), donkey anti-guinea pig-555 (1:500), donkey anti-rat-555 (1:500), donkey anti-chicken-555 (1:500), donkey anti-rat-647 (1:500), anti-guinea pig-647 (1:500), donkey anti-rabbit-647 (1:500), donkey anti-mouse-647 (1:500), phalloidin (Invitrogen A12379, 1:250).

Sequences used for hybridization chain reaction (HCR) are listed in Supplementary Table 3. HCR probes were designed as described in ^48^.

Images of immunohistochemical stains were acquired using a Leica SP8 confocal microscope. Confocal image stacks were processed with ImageJ and Adobe Photoshop (v23.4.1). All figures were made in Adobe IIIustrator (v29.5.1).

### Statistical analysis

The difference of the number of Dm8s present between WT and sev.lz adult brains was compared by two-sided, unpaired Student’s *t*-test (data fit assumptions of normality) in Fig. 5u. Data are shown as mean ± s.d. with individual values. Statistical significance was shown as *P*<0.05. Data analyses and plot were made in Prism (v10.4.2).

## Extended Data

**Extended Data Fig. 6:**
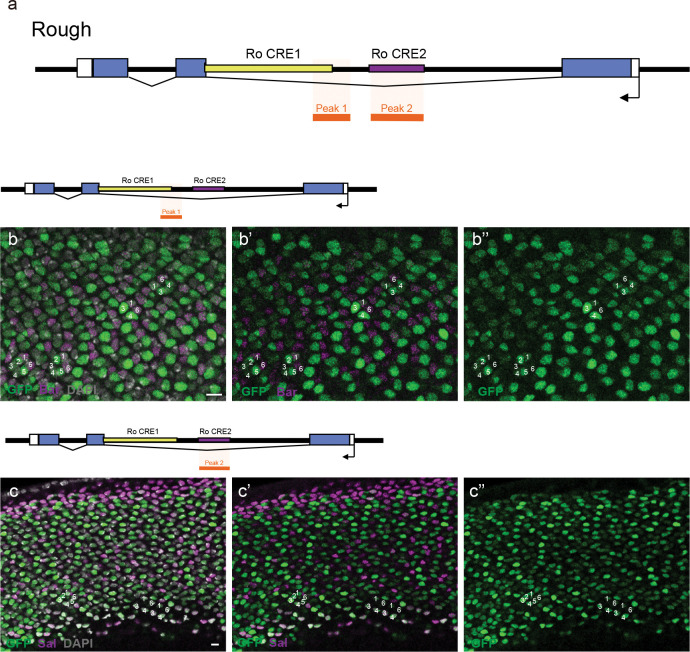
Rough CRE testing in *Drosophila*. **a**, Overview of the *Rough* gene in the *Drosophila* genome. Two Rough CREs were tested in intron 2, chosen partly based on single cell ATAC Seq data from ^49^, with peaks shown in light orange. **b,** Ro CRE 1 driving expression of GFP (green) in the *Drosophila* 3^rd^ instar larval eye disc. Expression was variable in PRs 1–6, with Bar (magenta) expression shown in **b’** and GFP expression alone in **b”**. **c,** Ro CRE 2 driving expression of GFP (green) in outer PRs 1–6, with Sal (magenta) labeling R3/4 in **c’.** GFP expression alone is shown in **c”.** Scale bar, 5μm.

**Extended Data Fig. 7:**
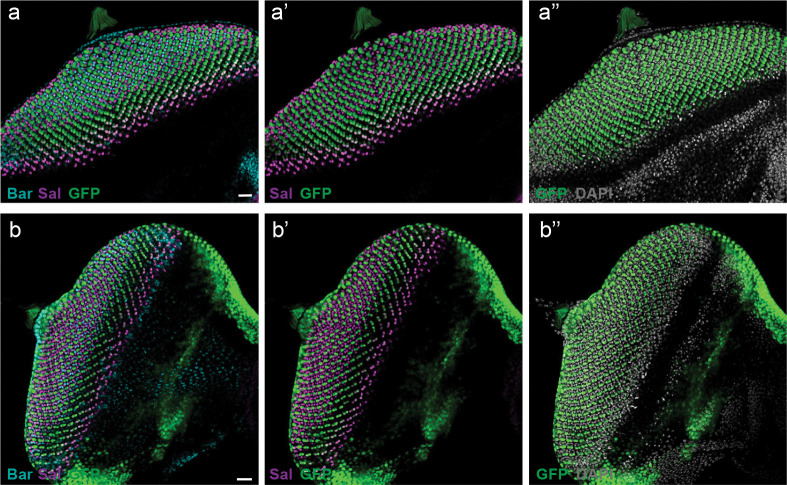
Two R3/4 CRE candidates in *Drosophila*. **a-a”**, R55F01 from the CG14509 locus drive reporter expression in R2/5 and R3/4. GFP (green) labels R2/5 and R3/4. Sal (magenta) labels R3/4. Bar (cyan) labels R1/6. **b-b”**, R86F02 from the Klumpfuss (Klu) locus drive reporter expression in R3/4. GFP (green) labels R3/4. Sal (magenta) labels R3/4. Bar (cyan) labels R1/6. All nuclei are labeled with DAPI (gray). Scale bar, 20μm.

**Extended Data Fig. 8:**
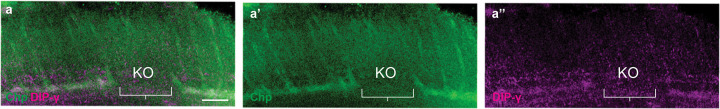
The effect of R7 loss on Dm8 retention in butterfly brains. **a**, CRISPR-induced mosaic knockout of *Lz* results in the loss of R7 axons and DIPγ expression in the *Vanessa* medulla. The *Lz* KO region (indicated by bracket) lacks Chp (green, **a’**) and DIPγ (magenta, **a”**), which are visible in adjacent WT regions. Scale bar, 10μm.

**Extended Data Fig. 9:**
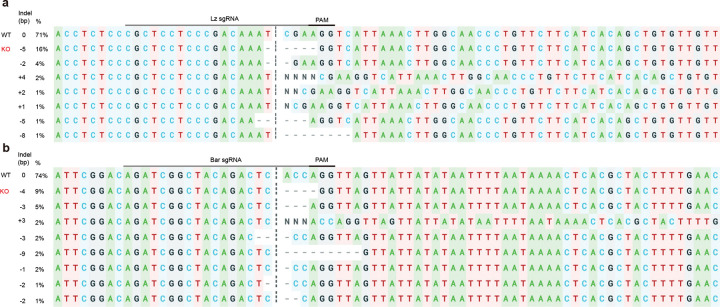
Genotyping validation of CRISPR-induced mosaic mutations. Sanger sequencing showing indels at the *Bar* (**a**) and *Lz* (**b**) target sites in *Vanessa* using Synthego ICE analysis. Predicted cut sites are indicated by dotted lines.

## Supplementary Material

Supplement 1
