# Supplementary material for: Sensory receptor expansion and neural accommodation in butterfly color vision
Source: bioRxiv. 2025 Oct 31:2025.10.30.685642. Preprint. [Version 1] doi: 10.1101/2025.10.30.685642 (PMC12636425; doi:10.1101/2025.10.30.685642)
Supplement: Supplement 1 [file NIHPP2025.10.30.685642v1-supplement-1.pdf]

bioRxiv preprint doi: https://doi.org/10.1101/2025.10.30.685642; this version posted October 31, 2025. The copyright holder for this preprint (which was not certified by peer review) is the author/funder, who has granted bioRxiv a license to display the preprint in perpetuity. It is made available under aCC-BY 4.0 International license.

Supplementary Table 1: Primers and sgRNA guides used in this study

1. CRISPR sgRNA	
Gene name	Sequences
Lozenge	TCTAGCACGTCGCCCAGGGC
	CGCTCCTCCCGACAAATCGA
Bar	TATACTCGACGCTGCGCCGA
	AGATCGGCTACAGACTCACC
2. CRISPR screening primers	
Gene name	Sequences
Lozenge	CGGGTACGTGCTTCTAtcttatg
	CTTCAAGGTAGTGGCCCTGG
Bar	GAAGGACGGCGATGGTTAAT
	CGGGTACGTGCTTCTAtcttatg

Supplementary Table 2: Sequences used to produce antibody

Protein name	Sequences
VcBar	MTVQRDERDARAPRTRFMITDILDAAPRDLSAHRDSDSDRSATDSPGVKDDS<br>DDVSSKSCGGDASGLAKKQRKARTAFTDHQLQTLEKSFERQKYLSVQDRME<br>LAAKLGLTDTQVKTWYQNRRTKWKRQTAVGLELLAEAGNYAAFQRLYGGYW<br>AGVPAYPAQPTPTADLYYRQAAATAAAAASASANTLQKPLPYRLYPGAPLGGV<br>PPLGLGLPGPSAHLGSLGAPGLGALGYYAQARRTPSPDVDPGSPAPPPRSPR<br>EPSIEQRSDDEDDDETIHV
VcRo	MDKEIYPEKSKDTSIQTPRPSSPRKFFARIYGHLDQPKTKDKEQTQGSESDSCSD<br>VEIGDNEGRDSLSASPLWPPPPLPVCPRPILLPHQQLAFGAGLAAFLARRRRKESR<br>PRRQRTTFSAHQTLRLELEYARGEYVARARRCELASALSLSETQVKIWFQNRRAKD<br>KRIEKAHLDQQYRQLAAASGFFQPIFNSPYGPPPCPCLPVHSPPNLEK
VcLz	MHLPYESRGRRRREMAELEPWWLQSIIDETLGEHPDLVRTGSPDYMCSMLPQH<br>WRSNKTLPGGFKVVALGDVLDGTQVTVRAGNDENCSAELRNNTAVMKNRVAKF<br>NDLRFVGRSGRGKSFSLTITVSTTPPQVATYQKAIKVTVDGPREPRSKTIILGTNA<br>STHQIRTIGFQRPFISGSSMALREMEYKSSARSLRSLSHEEREYKTNANLPTEEN<br>TGNILGASEWNTGYPSSASVYPSYGPLQPAYYKPDPTIHIPAVLPEIPLGHTSDYS<br>GFQSGSTGFVKGSPSGASGTLTDLNTPAMTTQRYDPNYYNSWPANSYNYQYNN<br>INNNPACLQSHTPYINPNPQMILPNLTYSTVNQNQIHVHLHSSSDKYNLEQYMPS<br>EIKINDIDGGISITTDLPGVGEPSGLVQTCESADDVKNGLYGSGSQEVWRPY
VcChaoptin	MSLMAIIKFGYTLIVVTFILMIWASLARALELHVDTGHPPCLFQALCTCSKPAGDL<br>GIVTCKHVPILRVPAAVNSSKVFTLQLTGNKIRDLEPHFFQATGMYRLAINQNPL<br>ESIQDEAFYGLDNTLWELELKQDRLTSVPSRALRYLQKLRLLDLTGNEITEITGD<br>NWLGLENSLQTLILSDNSIATLPLDAFSGLLILETLDLHGNHLSVIDSGVFRDGMS<br>RLSKLLLGDNQLTLIPYEELSPLRQLRHLDLSNNLIKQVPPAHDLNGVKLSLDYLK<br>LDQNNIKILLPGSFKYFNILNTTSLNGNPIFTIREDAFRNAKIKTLSLRDCGVTELSP<br>ASFAGLENSLQNLDLSENNLTMISKFMLNKLDSLRFLNLRENKVDTNLLATNNPS

bioRxiv preprint doi: https://doi.org/10.1101/2025.10.30.685642; this version posted October 31, 2025. The copyright holder for this preprint (which was not certified by peer review) is the author/funder, who has granted bioRxiv a license to display the preprint in perpetuity. It is made available under aCC-BY 4.0 International license.

	EYSTTPSVNNFQYKLFYLDISGSSSLEMSLQDVRRMRSLRYLAVSKLIRRSITSE<br>DFLEFGVELEDLKIIGSTINRIEASAFQHVRTIKTLDLSENNIDFIDPFAFAELHSLT<br>TLKMANGLADSVKILPFEPLKALIELEYLDLSNNKLKNVPDTSFHFMYKLKTLNL<br>QDNLIDQFSKGTLQSDIHRELESVCLSLNQLQRIDQHTFVNLRELQEILIEDNLIE<br>TVYRRSFTSLDNLKVIRLRGNIITEISEESFQNLPALKELDISFNQLETFKFSIFDQ<br>VGSATALKVNVSYNRIISLTDSNAPSFFTSNFYPPPKAQRLVSEDPSPLRIERGL<br>GTVSVNIRVLDFSHNNISYIAPYYFRHADLTLSELYLSYNLIRNVTREVFGSMLM<br>LQYLDLSHNQIFHMEYDCFKKVKNLQIIDLSHNHLIELPVEVFHDMQALTSVDLS<br>DNNIKNLADNLIISPSLERLDLSDNDLSRIPTNSLSPAAAINLVELDLSGNTIPAVAI<br>ADLVQRFRHEPSEYWLEEPDYSDEYMYHTARRDHPRVLHRKIQYPQNVLYKSL<br>AWLDLSDNHLVRVESGSFTALPKLLWLDLSMNTPFNNGERGSSIFKGLERRLSH<br>LGLKNVSLTSVPSMPLPKLKSLDLSYNNLPSVPTDITANLTRLRSLDLSYNDLTNV<br>PVATHSLSDLRWLSLSGNPISALMNTSMYGVSPRLEYLDVTHLKLSILEHGAFSK<br>MYGLRTLKISVNGNIRDFNIPKILTHNDALENLYLQIDNTQVDLSKEMLGLLPSKLN<br>NITITGRALKFLSQNVLKGVQSQTLTLTVYNTSIEEIENEVFWRPGRIKNLTLDLRD<br>NMIARVPNPARHEWPGVPNSLFLHNIYVAGNPLQCDCRIGWVQAWDRKRRQYL<br>CDGPSDCIATRDDVRFAKCPSHYNRTFSDVIAKDLDCSWSKGFFRSPNIFIIITMS<br>ILTYLYI
VcDIP-γ	MRSEKSCFWSMLQALALLPTMALKDSQTTTKKFSEEPAFERPIGNHTFFLGREAV<br>LGCAVTNLGKHKVGWLRAEDQTVLTMHERAVLGSRYAVSLDAPRTWQLRIRPLRA<br>EDRGCYMCQINTQPTMTWQIGCIDVFVPPDIVSDDTSGDVSVQELENATLTCKATG<br>HPPPKITWRREDHEPILLKKPSSRDFDKVESYVGSSMPLWRVDRRQMGAFLCIAS<br>NDVPPAVSKRITLNVNFAPTVKVPNQLLGAPLGTDVKLKCYVEAYPNTINYWIKNRG<br>EMLLDGPKYTIREEKTSYKVSMWLTIRQFSKSDIGTYNCVSTNSLGKSEGTLRLYEI<br>KLNSYSEDFSNQISVAGGLTEAAKGNSDDKFRIERTLLLMLFCIVFMLT

bioRxiv preprint doi: https://doi.org/10.1101/2025.10.30.685642; this version posted October 31, 2025. The copyright holder for this preprint (which was not certified by peer review) is the author/funder, who has granted bioRxiv a license to display the preprint in perpetuity. It is made available under aCC-BY 4.0 International license.

Supplementary Table 3: Sequences used to design HCR probes

Gene	Sequences
Vc_RunxB	MHLSSEVSSTTNGLGHEAPSSSYGNALPSQPLTAELLAERTLEGLLADH<br>PGELVKTGCPHVVCTVLPPHWRSNKTLPVAFKVVALGDVGDGTLVTVR<br>AGNDENCSAELRNCTAVMKNQVAKFNDLRFVGRSGRGKSFTLTIMLATS<br>PPQVATYQKAIKVTVDGPREPRSKTRHHGFHPFHFGPRFAPDPLMGSL<br>PFKLSGIAHQLAGLPGGEWGALARHYPPPLLPTHAHFTPHVLPPAHAQH<br>HTVPPPPHETDLASDNPNTMHPHSTTSPVNSRPSSPQDDDDISVTASS<br>SPPPDDRPGAFTSVTRPRKPLQPLPAPSSLFHSALAAQLFLNSPLLPTP<br>PSWLYSQLYGGYDWWLRPPPQQDDSNSSPDREEEAGSTASSSTKKRP<br>ASPEWTDQGVRTRSKSLITSERRPVDVWRPY
Vc_Dpr11	MCACAWRLHAFTAALVLAAHLIKVLSCGETGGGGGTRARRYVGLYTGP<br>YFDPSAPNNITAQLGTHAYLPCKVRQLSNKSVSWIRRRDAHILTVDRFTF<br>IADERFQAFLVEATDTWTLQVKYVQARDAGVYECQVGTEPKMSHFVQL<br>NVVVPKIEIVGESDLYVKAGSTVSLKCVITQALEEPAYIFWYHNDERVLNY<br>DRSLVEIRMERLAPDTTIGNLIIYNPRREDSGNYSCSPSNLDSASVVLHV<br>LSGEQPAAMQHGNGAAPTRARALLLSAAAALAAPRRSRVLALLALALILP<br>LVAVPRRRLTKIASAARRLARTVDPVKYRVLRVRDACPRTSPIRYKLLLSL<br>EPEGSDDGASSFNILSIISRRVHRWDLSTSGDRLRVIIYFRRRLRSGAAS<br>ARRRRGVGAASARRRRGVGAQARPAFVKLYDEQ
Vc_Cut	MHPTGAASLPAAPEAEVQAMHSMDWLFKKERIYLLAQFWQQVIKSTTQ<br>PINTRATLAEKEVTTLKEQLATTSPTPLQATVPPKTNGSHIESTRDQATET<br>RIPERFSPDIKEEKRRSPDIDEDIEQKIEMAATARSNSNSSRSSPVVNQS<br>SSLENELAAKEKEIAQLVEDVRRLQASLSALQEAHAQQLQRLEERLDEK<br>KQHIARLEARLDTQRDYDEIKREISMLRFSELGPTERPHSHTLPKDIFGS<br>QNHLLRSMDLGPNERVERKEALRSPAAPAHRDSSAERERSTERRDTG<br>GDEWPSTPPPLNNNTTHHNNNGPVPLPLPPPSPFRFEEHRPYRFAED<br>MGPLPPGALVGRLGDSLIPKGDPMEARLQEMLRYNMDKYANSNLDTLH<br>ISRRVRELLSVHNIGQRLFAKYVLGLSQGTVSELLSKPKPWDKLTEKGR<br>DSYRKMHAWACDEAAIMLLKSLIPKKVGTKDGSAGTGFGRPEGEGDER<br>LAHMLNEASHLMKTPTGQPNNDDSRSNEDSSSPRTQCPSPFSKDSSQ<br>NRRLKKYENDDIPQEKVVRIYQEELAKIMTRRVEDMRHNREGFPGVFPP<br>FFSGGMPPHMERPPEDIRMALEAYHRELAKIQPGGNIPNLHNMPGMPP<br>FPNLLALQQQALQAQNQHMNGSGAVQDLSLPKDKNTKINGLTDSDKDR<br>SMDAEEAIRHAGSAFSLVRPKLEPGQQSTGSSASSPLGNAILPPAITPND<br>DFSNSAAASPLQRMASITNSLISQPPNPPHHAPPQRSMKAVLPPITQQQ<br>FDLFNNLNTEEIVKRVKEALSQYSISQRLFGESVLGLSQGSVSDLLARPK<br>PWHMLTQKGREPFIRMKMFLEDDNAVHKLVASQYKIAPEKLMRTGNYS<br>GAPPCPPNMNKPMPPTQKMISDATSLLSKMQQEQLLSSGHLGHLGQPT<br>PLLLTPPGFPPHHAVTLPPQHHDNNNKERKPPPPPQPHHQPPVMRGLH<br>QHMSPSVYEMAALTQDLDTQTITTKIKEALLANNIGQKIFGEAVLGLSQG<br>SVSELLSKPKPWHMLSIKGREPFIRMQLWLSDAHNIDRLQALKNERREA<br>NKRRRSSGPGQDNSSDTSSNDTSEFYHSSSPGPTSGVPSAKKQRVLF

bioRxiv preprint doi: https://doi.org/10.1101/2025.10.30.685642; this version posted October 31, 2025. The copyright holder for this preprint (which was not certified by peer review) is the author/funder, who has granted bioRxiv a license to display the preprint in perpetuity. It is made available under aCC-BY 4.0 International license.

	SEEQKEALRLAFALDPYPNTPTIEFLAAELGLSTRTITNWFHNHRMRLKQ<br>HAPHGLPAEPPARDQTAAPFDPVQFRLLLNQRLLELQKERMGLAGVPL<br>PYPPYFAANSNFAALIGRGLLPPEERVKDPTSGLDLSMPLKREPDGDDF<br>EDDDVESNLGSDDSLDEESKNEPKAASTPASRSNRRKPAAPQWVNPD<br>WQDEKPRNPDEVIINGVCVMRSDDFRREAEETVRVEPSPVPREPSPAA<br>SPASRASRASPASLASHARSLARTPDVLPEDKIKTEAEDDRWDY
Vc_Side_VII	MIEIIMPLALCRFIASAAILGVAVATTARNAPLTQVVAVAGEPVYLPCDVAT<br>QDEDDAVLLVLWYREDLGTPIYSVDAREKDFGVAERWSDESVFASRAYF<br>LPERRPAELGVDRVRATDQGIYRCRVDFKQAQTRNSRVNLTVIVPPNKM<br>VITDDKGDIKQAIVGPYVEGDTFTLKCDVSGGRPRPWVKWFRNELETD<br>TPATTLQGTSVRGVLRVGPLTRADVRATLTCRASNHLRAHPIETTLTLDM<br>NFPPLNVHILGSNQPLSASRRYDLLCQSSGSRPPASITWWKNGHRINNA<br>KETLSTDGNTTTSTVSIQLNKADAGAKLACRASNPQMPSIPAMEDDWLL<br>DIQYVPETVVRLGTNLDPSNIREGSDVYFDCIIKAHPYVYKVEWRHNGK<br>TLHHNVGQGIIISNQSLVLQGVGRKTAGNYTCVGFNAEGDGESKAFSLN<br>VLYAPTCRSSQQRVHGVAKQERAHITCHVDANPPEVSFRWTFNNTANS<br>NEVSDTYVSRSGTSSTVTYTPHTEMDYGTLLCWAHNRIGKQRVPCVYH<br>IIAAGRPDQVHNCTVVNASLTSFGVRCSEGFNGGMPQSFLLEVREIVTQ<br>EIVANVSSAVPRFTAVGLVPGRTYAAAVLAYNAKGRGDPYPLRASTLRPP<br>EKHHVHDKSLDLPRTAFQMSGAMSAAVGGGGVLMVVLVLFMIAVRQRC<br>AKRKPRSAPPPSQPASPDKLREKDDSESDDRNPDIIPSDTDHNMQMDY<br>YRQQQVSTISPPSSRARGSIAGVRGGLPAYCALRTAPPSDSTPPSGIPP<br>PAGFASGSCTLPRNAGTANERCIPMQHLRTLPRAHPHPHPHPHVHAHA<br>HPHPPRDTPL
Vc_Svp	HTRAPPRSHPATATGIITHGALVYGGVGGPGLCPPRASDLPPLELGFRG<br>SWREDELLPSTPASQAASTQSGSSANDKGQNVECVVCGDKSSGKHYG<br>QFTCEGCKSFFKRSVRRNLTYSCRGNRNCPIDQHHRNQCQYCRLRKC<br>LKMGMRREAVQRGRVPPTQPAGLALPGQFALANGDPAAGLNSHPYLS<br>SYISLLLRAEPYPTSRYGQCVQPTNVMGIDNICELAARLLFSAVEWARNI<br>PFFPELQVTDQVALLRLVWSELFVLNASQCSMPLHVAPLLAAAGLHASP<br>MAADRVVAFMDHIRIFQEQVEKLKALHVDSAEYSCLKAIVLFTTDACGLS<br>DVPHIESLQEKSQCALEEYCRSQYPNQPTRFGKLLLRLPSLRTVSSQVI<br>EQLFFVRLVGKTPIETLIRDMLLSGSSFSWPYMATM

Supplementary Table 4: Six of the 61 lines from the Janelia FlyLight collection that were evaluated as potential R3/4 enhancers.

Number	Stock no.	Gene name
R65A05	*39329*	*abl*
R86F06	*46824*	*klu*
R86F02	*46823*	*klu*
R55F01	*39124*	*CG14509*
R22E03	*61641*	*pkcdelta*
R24C05	*49313*	*argos*
